# Ketogenic Diet Decreases Alcohol Intake in Adult Male Mice

**DOI:** 10.3390/nu13072167

**Published:** 2021-06-24

**Authors:** María del Carmen Blanco-Gandía, Francisco Ródenas-González, María Pascual, Marina Daiana Reguilón, Consuelo Guerri, José Miñarro, Marta Rodríguez-Arias

**Affiliations:** 1 Department of Psychology and Sociology, University of Zaragoza, C/Ciudad Escolar s/n, 44003 Teruel, Spain; mcblancogandia@unizar.es; 2 Unit of Research Psychobiology of Drug Dependence, Department of Psychobiology, Facultad de Psicología, Universitat de Valencia, Avda. Blasco Ibáñez, 21, 46010 Valencia, Spain; francisco.rodenas-gonzalez@uv.es (F.R.-G.); maria.pascual@uv.es (M.P.); marina.reguilon@uv.es (M.D.R.); jose.minarro@uv.es (J.M.); 3 Department of Molecular and Cellular Pathology of Alcohol, Principe Felipe Research Center, C/Eduardo Primo Yúfera 3, 46012 Valencia, Spain; guerri@cipf.es; 4 Department of Physiology, School of Medicine, Universitat de Valencia, Avda. Blasco Ibáñez, 15, 46010 Valencia, Spain

**Keywords:** ketosis, alcohol, ketogenic, ketone, adenosine, dopamine

## Abstract

The classic ketogenic diet is a diet high in fat, low in carbohydrates, and well-adjusted proteins. The reduction in glucose levels induces changes in the body’s metabolism, since the main energy source happens to be ketone bodies. Recent studies have suggested that nutritional interventions may modulate drug addiction. The present work aimed to study the potential effects of a classic ketogenic diet in modulating alcohol consumption and its rewarding effects. Two groups of adult male mice were employed in this study, one exposed to a standard diet (SD, *n* = 15) and the other to a ketogenic diet (KD, *n* = 16). When a ketotic state was stable for 7 days, animals were exposed to the oral self-administration paradigm to evaluate the reinforcing and motivating effects of ethanol. Rt-PCR analyses were performed evaluating dopamine, adenosine, CB1, and Oprm gene expression. Our results showed that animals in a ketotic state displayed an overall decrease in ethanol consumption without changes in their motivation to drink. Gene expression analyses point to several alterations in the dopamine, adenosine, and cannabinoid systems. Our results suggest that nutritional interventions may be a useful complementary tool in treating alcohol-use disorders.

## 1. Introduction

Motivation to seek drugs of abuse and highly palatable foods is regulated by the reward system [1]. Previous studies have shown psychological and biological commonalities between palatable food intake and drug addiction [2,3] and recent studies have indicated that nutritional habits are an important modulating factor in the development of cocaine [4,5] and alcohol addiction [6,7]. Palatable diets change metabolism and the reward system by increasing vulnerability to the rewarding effects of psychostimulants and depressants, such as cocaine and alcohol [4,6,8], but little is known about the protective effects that nutrition could have in the development of drug addiction. In a recent study, a high-fat diet was shown to reduce relapse into cocaine seeking in adult male mice [5]. However, the diet employed in those studies was rich in saturated fats and sugar, producing metabolic syndrome and obesity when the HFD administration was continuous. and therefore, it cannot be recommended as a drug-addiction combined therapeutic. This metabolic syndrome is avoided when the administration of HFD is intermittent, although the accelerated extinction and the blockade of reinstatement is maintained [9]. Nevertheless, this contribution opened a new gateway to focus on nutrition as a possible complementary treatment in the field of drug addiction. For this reason, we considered the classic ketogenic diet (KD), a diet still high in fats, to prevent the development of escalation in alcohol consumption.

Today we can find several eucaloric protocols like the medium-chain triglyceride diet (MCT), the modified Atkins diet (MAD), the low glycemic index treatment (LGIT) or the classic ketogenic diet (KD). The KD is a high-fat, low-carbohydrate, and protein-balanced diet that induces a different metabolic state, in which ketone bodies are used as the main energy source [10,11]. The decrease in carbohydrates reduces the amount of glucose, which ceases to be available as the main source of energy [11]. This is where the body begins to use fat storages, breaking down fatty acids and creating ketone bodies in the liver (e.g., acetoacetate, β-hydroxybutyrate or βOHB to produce ATP, adenosine triphosphate) [11,12]. The rise in blood ketone bodies appears as a response to low glucose levels, known as metabolic state ketosis. Ketosis can be achieved in two ways: through diet (nutritional ketosis) or through fasting. In the present work we will focus on nutritional ketosis.

Ketosis is not new. Evolutionarily, humans have spent a good part of their existence in ketotic state (especially in winter, where carbohydrates such as fruits and vegetables were limited). Moreover, ketone bodies play a very important role in the normal fetal brain development [13,14], as breast milk is high in fat and medium-chain fatty acids, inducing a ketotic state in the new-born [15,16]. The KD is neuroprotective [17], since ketone bodies cross the blood-brain barrier without difficulty and increase metabolic efficiency by improving mitochondrial function [18,19]. It reduces oxidative stress, with antioxidant and anti-inflammatory effects, inhibiting inflammatory markers such as interleukins and tumour necrosis factor alpha [20]. However, the mechanism through which a KD is beneficial is still under study. The KD has been used successfully for different disorders such as epilepsy [21,22], Alzheimer’s and Parkinson disease [23,24,25,26,27], brain cancer [28,29], autism [30,31], and amyotrophic lateral sclerosis [32]. Based on all of these studies, we can hypothesize that ketones induce a normalization process when metabolism functioning is dysregulated, which can account for its beneficial effects on all of these neurological disorders.

Diet and nutrients can exert great changes in neural plasticity, modifying circuits and normalizing their function [33,34,35]. One of the most important KD neural mechanisms is its modulation of ATP-sensitive potassium channels and increase of GABAergic and purine neurotransmission such as adenosine [36,37]. The KD activates adenosine receptors, which are the basis of its therapeutic effects on diseases such as epilepsy, as it inhibits the excitability of neurons [38,39,40]. Adenosine regulation is closely linked to the dopaminergic action, as its receptors are colocalized on GABAergic neurons together with dopamine D2 receptors, suggesting that modifying one may lead to the regulation of the other [41]. In fact, there is an antagonistic interaction between the heterodimers of the adenosine A1―Dopamine D1 vs. adenosine A2A―dopamine D2 receptors [42]. Although some of the results are controversial, there is evidence indicating that adenosine mediates the response of drugs such as opiates, cannabinoids, and psychostimulants [43,44,45,46]. For example, cocaine self-administration (SA) induces an upregulation of A2 receptors and a downregulation in D2 receptors as a compensation, a situation that is reversed with abstinence [47].

To date, only a few studies have shown the protective effect that a KD can have on drug addiction. Martínez and co-workers [48] showed that animals on a KD showed decreased cocaine-induced stereotypies and sensitization in male and female rats, suggesting that the KD modulates the dopaminergic system. Regarding alcohol, KD has been reported to decrease alcohol withdrawal symptoms in rats [49] and mice [50], as well as reduce alcohol consumption in rats [51]. The recently published work by Wiers and co-workers [51] combines a preclinical and a clinical study. In relation to the clinical study, Wiers and co-workers [51] also found that people with alcohol-use disorder who were on a KD had fewer withdrawal symptoms and required fewer medication the first week of detoxification than those following a standard American diet [51].

Therefore, the main aim of the present study is to evaluate whether a KD could modulate the rewarding effects of alcohol by acting through the adenosine-dopamine binomial. To achieve this objective, the behavioral effect of a KD on the oral ethanol SA was studied. Additionally, we evaluated the main changes in dopamine and adenosine receptor gene expression in the striatum, taking into account that diets and nutrition can disrupt the normal plasticity in the dorsal striatum [52]. Moreover, given the implication of the cannabinoid and opioid systems in the rewarding effects of not only alcohol but also high-fat foods [53,54], we included the analysis of the CB1r and Oprm expression.

## 2. Materials and Methods

### 2.1. Subjects

This study was performed with 47 OF1 male mice (Charles River, Écully France), which were housed under standard conditions (21 ± 2 °C) in groups of 4 (cage size 28 × 28 × 14.5 cm). Animals were 21-days old on arrival at the laboratory but initiated the experimental feeding condition on PND 42. Lights were turned on from 8:00–20:00, and food and water were available ad libitum. All procedures involving mice and their care complied with the national, regional, and local laws and regulations, which are in accordance with Directive 2010/63/EU of the European Parliament and the council of 22 September 2010 on the protection of animals used for scientific purposes. The Animal Use and Care Committee of the University of Valencia approved the study with the code 2019/VSC/PEA/0065 on 23 March 2019.

### 2.2. Drugs Treatment

Absolute ethanol (EtOH) (Merck, Madrid, Spain) was dissolved in water using a *w*/*v* percentage, i.e., a 6% (*w*/*v*) EtOH solution equivalent to a 7.6% (*v*/*v*) EtOH solution. Saccharin sodium salt (Sigma, Madrid, Spain) was diluted in water.

### 2.3. Apparatus and Procedure

#### 2.3.1. Experimental Design

In the present study we employed two animal sets. In the first set, animals in the standard diet (SD, *n* = 15) group received the standard diet throughout the procedure, while animals in the ketogenic diet (KD, *n* = 16) group received the ketogenic diet from young adulthood, PND 42, until the end of the experiment. All the animals began the training phase on PND 49 and the 6% EtOH consumption phase on PND 66, when mice are already considered adults. Bodyweight and beta-hydroxybutyrate (βOHB) blood levels were measured every week throughout the study. During the SA procedure, animals had 1 h access to food per day. About 15% weight loss was produced by the food restriction schedule [55]. Brains were collected at the end of the SA for gene expression analysis.

**Figure 1:**
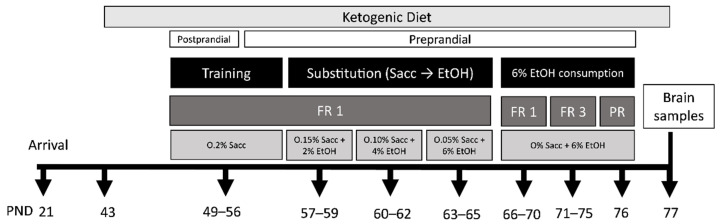
Experimental design for the 1st set of mice. FR, fixed ratio; PR, progressive ratio; PND, postnatal day; Sacc, saccharin; EtHO, ethanol.

In the second set, a total of 16 animals were used (*n* = 8 per diet). They had the same feeding conditions and initiated the diet conditions on the same PND as the first set, but without any behavioral manipulations. In order to test only the effects of KD, animals in this second set were not exposed to alcohol. They were euthanized for brain gene expression analysis on PND 77, as with the first set. A detailed description of the experimental procedure is shown in Figure 1.

#### 2.3.2. Feeding Conditions

Two different types of diets were used in this study. The SD group was fed with the standard diet (Teklad Global Diet 2014, 13 kcal % fat, 67 kcal % carbohydrates and 20% kcal protein; 2,9 kcal/g) and the KD group with a ketogenic diet (TD.96355, 90.5% kcal from fat, 0.3% kcal from carbohydrates and 9.1% kcal from protein; 6.7 kcal/g). The different diets were supplied by Envoi Teklad Diets (Barcelona, Spain).

#### 2.3.3. Ketosis Status: β-Hydroxybutyrate Blood Levels

Blood β-hydroxybutyrate was measured weekly from the tail vein with an On Call GK Dual monitor and ketone test strips (ACON Laboratories, Inc., San Diego, CA, USA).

#### 2.3.4. Oral Ethanol Self-Administration

Following the previously published protocol [55], eight modular operant chambers (Med Associates Inc., Georgia, VT, USA) and Med-PC IV were employed to carry out the oral EtOH SA. These cages contain two small holes with photocells that register nose-poke responses. Active nose-pokes activated a 0.5 s stimulus light and buzzer beep, which were followed by the delivery of 37 μL of EtOH, followed by a time-out of 6 s. Inactive nose pokes did not have any effect. This protocol consists of three phases: training phase, saccharin substitution phase, and 6% EtOH consumption phase.

- Training phase (8 days)
  In the training phase, animals had to nose-poke into the active wholes to obtain 37 μL of saccharin (0.2% (*w*/*v*)). To facilitate learning acquisition, two days before beginning the training, chow was restricted to 1 h/day and water was suspended for 24 h before the first session. Only during the subsequent 3 days, 1 h before initiating the operant session animals had access to food but not to water (postprandial). On the subsequent four days and during the course of the experiment, to avoid EtOH intake due to thirst, water was available anytime and food was available for 1 h after each training session (preprandial).
- Saccharin substitution (9 days)
  In this phase, saccharin percentage was progressively reduced as the EtOH concentration was gradually augmented [55,56]. Animals had access to each combination for three consecutive sessions (0.15% Sac −2% EtOH; 0.10% Sac −4% EtOH; 0.05% Sac −6% EtOH).
- 6% Ethanol consumption (11 days)
  This phase evaluates the number of active nose-poke responses, the 6% EtOH (*w*/*v*) intake and motivation to obtain it. First, animals were exposed to 5 days of fixed ratio 1 (FR1) sessions, in which the number of effective responses on the active nose-poke and EtOH consumption (all) was measured. After each session, the remaining fluid in the receptacle was collected and quantified with a micropipette. Following the FR1 sessions, animals were exposed to the fixed ratio 3 (FR3) schedule for 5 days, where they had to respond three times with an active nose poke to obtain one EtOH reinforcement. To set the breaking point for each animal, which is the maximum number of active nose-pokes the animal is capable of accomplishing to obtain one reinforcement, a progressive ratio (PR) session with a 2 h duration was carried out. The response requirement to achieve reinforcements increased corresponding to the series: 1-2-3-5-12-18-27-40-60-90-135-200-300-450-675-1000. The breaking point that the animal achieved was calculated based on this scale, which defines the animal’s motivation toward EtOH consumption.

#### 2.3.5. RNA Isolation, Reverse Transcription, and Quantitative RT-PCR

After the PR session, mice were euthanized by cervical dislocation, and their brains were extracted and striata dissected. Brain tissue samples were immediately stored at −80 °C until the qRT-PCR assay was performed.

Following the manufacturer’s protocol, the Tri Reagent Method (Sigma-Aldrich, St. Louis, MO, USA) was employed to isolate the total RNA from the striatum. Reverse transcription of 1 mg of total RNA was performed via the Transcriptor First Strand cDNA synthesis kit (Thermo Fisher Scientific, Madrid, Spain). Amplification of the target and housekeeping (b-glucuronidase) genes was completed employing the Taqman Gene Expression Master Mix (Thermo Fisher Scientific, Madrid, Spain) in a LightCycler 480 System (Roche Diagnostics, Madrid, Spain). The assay codes of the primers used were Mm02620146 and Mm00438545 for dopamine receptors 1 and 2 (DrD1, DrD2), Mm01308023 and Mm00802075 for adenosine receptors A1 and A2 (ADORA1, ADORA2a), and Mm01188089 and Mm00446953 for cannabinoid receptor 1 (CB1r) and opioid receptor µ (Oprm), respectively. Data were analyzed using the LightCycler 480 relative quantification software and normalized to the amplification product of b-glucuronidase.

### 2.4. Statistics

Data relating to body weight and βOHB were analyzed by a mixed ANOVA with one between-subjects variable diet (with 2 levels, standard diet (SD) and ketogenic diet (KD)) and a within variable PND with 6 levels: Baseline and Weeks 1–5. 

Regarding EtOH SA, a two-way ANOVA was performed with the variable diet (standard or ketogenic) as a between variable and days (5 levels for FR1 or FR3) as a within variable. To analyze breaking point values in the progressive ratio, a Student’s test was performed. The gene expression data were analyzed by a two-way ANOVA with two between variables, diet (with 2 levels, standard diet (SD) and ketogenic diet (KD)) and EtOH (no-alcohol self-administration (NO-SA) and alcohol self-administration (SA). Bonferroni post-hoc tests were also analyzed. Data are presented as mean ± SEM. Analyses were performed using SPSS v26 (IBM SPSS Statistics for Windows, Version 26.0. IBM Corp, Armonk, NY, USA).

## 3. Results

### 3.1. Increased β-Hydroxybutyrate (βOHB) and Bodyweight in Mice Fed on KD

**Figure 2:**
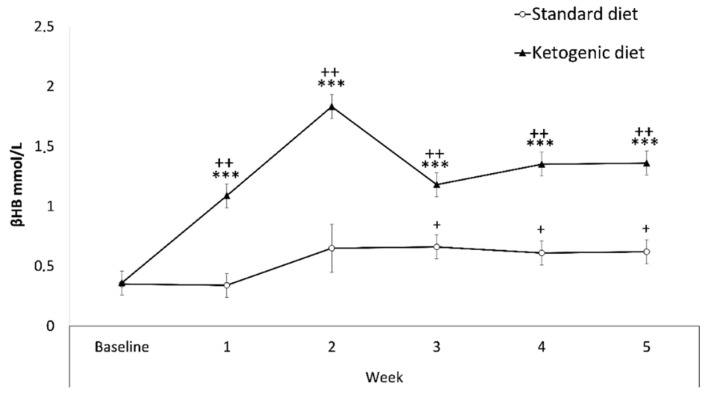
Weekly β-hydroxybutyrate blood levels. Data are represented as the mean (±SEM) amount of βOHB measured weekly. *** *p* < 0.001 significant difference with respect to SD group. ++ *p* < 0.001; + *p* < 0.05 significant difference with respect to the baseline.

Results regarding βOHB blood levels (Figure 2) revealed a significant effect of the interaction week x diet [F(5145) = 12,899; *p* < 0.001]. The KD group showed increased levels of βOHB in comparison with the SD group in Weeks 1, 2, 3, 4, 5 (*p* < 0.001 in all cases). There were also significant increases within the KD group on weeks 1, 2, 3, 4, 5 with respect to the baseline (*p* < 0.001), as well as increases in the SD group in Weeks 3, 4, and 5 with respect to the baseline (*p* < 0.05), probably due to coincidence of food deprivation during the SA procedure.

**Figure 3:**
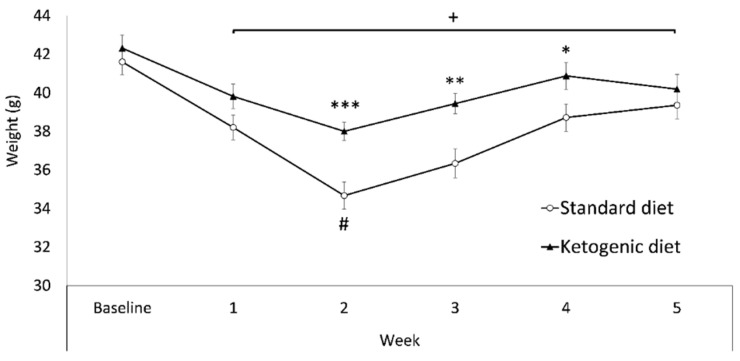
Weekly bodyweight. Data are represented as the mean (±SEM) bodyweight measured weekly. *** *p* < 0.001; ** *p* < 0.01; * *p* < 0.05 significant difference with respect to the SD group. + *p* < 0.05 significant difference with respect to the baseline. # *p* < 0.001 significant difference with respect to the rest of the weeks.

Regarding changes in bodyweight (Figure 3), the ANOVA revealed a significant effect of the variable diet [F(1,29) = 6.731; *p* < 0.05], as the KD group presented a higher bodyweight than the SD group (*p* < 0.05, SD = 38 gr vs. KD = 40 gr). There was also a significant effect of the variable week [F(5145) = 46.893; *p* < 0.001], as all weeks showed lower bodyweight than baseline (*p* < 0.001). In addition, during Week 2 (when the food deprivation for oral SA started) bodyweight was significantly lower than the rest of the weeks (*p* < 0.001). Finally, there was an effect of the interaction week x diet [F(5145) = 3.705; *p* < 0.01]. The KD group exhibited higher body weight with respect to the SD group on weeks 2, 3, and 4 (*p* < 0.001; *p* < 0.01, and *p* < 0.05 respectively).

### 3.2. Ketogenic Diet Decreased Oral Ethanol Self-Administration

**Figure 4:**
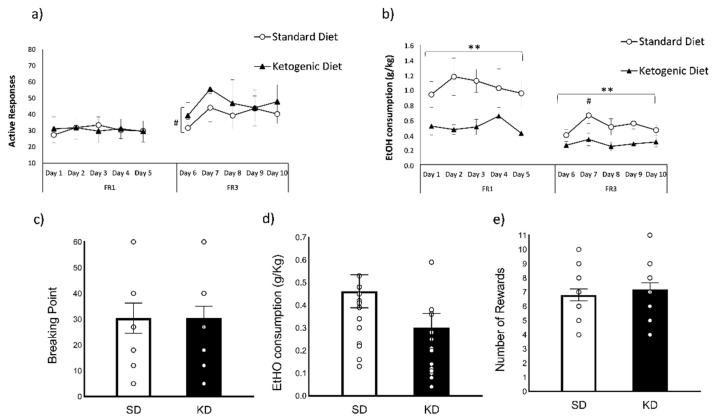
Oral EtOH self-administration (SD *n* = 15; KD *n* = 16). (**a**) The dots represent means and the vertical lines ± SEM of the amount of the number of active responses and (**b**) the volume of 6% EtOH consumption during FR1 and FR3 (in g/kg). (**c**) The columns represent means and the vertical lines ± SEM of breaking point values (**d**) the volume of 6% EtOH consumption (in g/kg) and (**e**) the number of rewards obtained during PR. ** *p* < 0.01, significant difference with respect to the SD group. # *p* < 0.05, significant differences with respect to Day 7.

Regarding the number of active responses during the FR1 schedule of EtOH SA (Figure 4a), the ANOVA did not show any significant differences between SD and KD. With respect to EtOH consumption (g/kg) during the FR1 schedule (Figure 4b), the ANOVA reported a significant effect of the variable diet [F(1,29) = 10.554; *p* < 0.01], as the KD group exhibited a decreased oral SA of EtOH with respect to the SD group (*p* < 0.01; KD = 0.51 ± 0.04 g/kg vs. SD = 1.04 ± 0.08 g/kg).

During the FR3 schedule, the ANOVA for the number of active responses (Figure 4a) showed a significant effect of the variable day [F(4116) = 2942; *p* < 0.05], as all mice exhibited lower number of active responses on Day 6 with respect to Day 7 (*p* = 0.076). With regards to EtOH consumption (g/kg) during the FR3 schedule (Figure 4b), significant differences were reported in the variable diet [F(1,29) = 8142; *p* < 0.01], since the KD group showed a decreased oral SA of EtOH (*p* < 0.01; KD = 0.28 ± 0.03 g/Kg vs. SD = 0.51 ± 0.04 g/Kg). There was also an effect of the variable day [F(4116) = 2638; *p* < 0.05], as all mice showed higher EtOH intake in day 7 with respect to day 6 (*p* < 0.05).

Regarding the progressive ratio (Figure 4c–e), there were no significant differences in the breaking point (t = −0.005 d.f. 29; *p* = 0.99), EtOH consumption (t = 1674 d.f. 29; *p* = 0.105), and the number of rewards (t = −0.616 d.f. 29; *p* = 0.54).

### 3.3. Gene Expression Analyses

#### 3.3.1. Ketogenic Diet Induced Increased Expression of DrD1 and DrD2 Gene Expression after Ethanol Self-Administration

**Figure 5:**
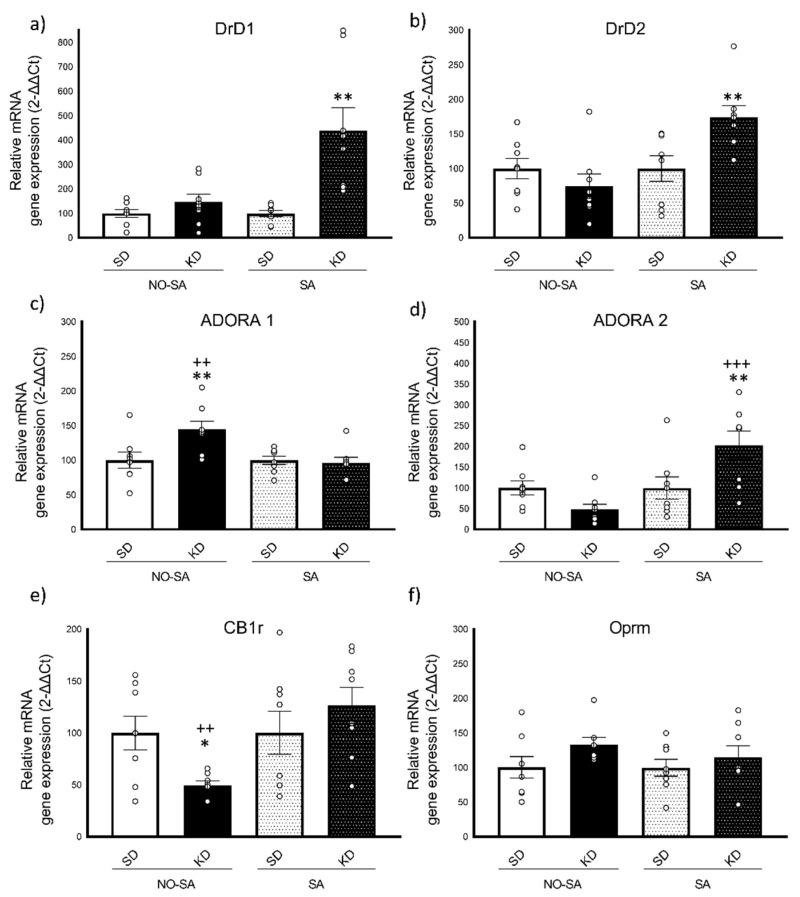
Real-time PCR Gene expression in the striatum (*n* = 8/condition). (**a**) Dopamine receptor D1 gene―DrD1, (**b**) Dopamine receptor D2 gene―DrD2, (**c**) Adenosin receptor A1 gene―ADORA1, (**d**) Adenosin receptor A2 gene―ADORA2, (**e**) Cannabinoid receptor 1―CB1r, (**f**) Opioid receptor µ―Oprm. The columns represent means and the vertical lines ± SEM of relative (2-ΔΔCt method) gene expression in the striatum of OF1 mice. * *p* < 0.05; ** *p* < 0.01 significant differences with respect to the corresponding SD group. ++ *p* < 0.01; +++ *p* < 0.001 significant differences with respect to their corresponding KD group.

For DrD1 gene expression (Figure 5a), the ANOVA revealed a significant effect of the variable diet [F(1,28 = 14.652; *p* < 0.001], EtOH [F(1,28) = 8.378; *p* < 0.01] and the interaction diet x EtOH [F(1,28) = 8.142; *p* < 0.01]. With regards to DrD2 expression (Figure 5b), the ANOVA revealed an effect of the variable EtOH [F(1,28) = 8.625; *p* < 0.01] and the interaction diet x EtOH [F(1,28) = 8.639; *p* < 0.01]. Exposure to a KD induced a significant increase in DrD1 and DrD2 expression in KD animals after the EtOH SA with respect to the rest of the groups (*p* < 0.001 in all cases).

#### 3.3.2. Opposite Changes in ADORA1 and ADORA2 Gene Expression in Response to Ketogenic Diet and Ethanol Self-Administration

For the adenosine receptor A1 gene expression (ADORA1, Figure 5c), the ANOVA revealed a significant effect of the interaction diet x EtOH [F(1,28) = 6.099; *p* < 0.05]. Mice in the KD group that did not perform SA (KD-NO SA) showed an overexpression of ADORA1 with respect to the corresponding SD group (*p* < 0.01) as well as with respect to the KD-SA group (*p* < 0.01).

Regarding the expression of the ADORA2 gen (Figure 5d), the ANOVA revealed a significant effect of the variable EtOH [F(1,28) = 10.587; *p* < 0.01] and the interaction diet x EtOH [F(1,28) = 10.615; *p* < 0.01]. After the EtOH SA, mice exposed to the KD showed a significant overexpression in ADORA2 with respect to their corresponding SD-SA group (*p* < 0.01) and KD-NO SA group (*p* < 0.001).

#### 3.3.3. Ketogenic Diet Decreases CB1r Gene Expression

With regards to CB1r gene expression (Figure 5e), the ANOVA revealed an effect of the variable EtOH [F(1,28) = 5.851; *p* < 0.05] and the interaction diet x EtOH [F(1,28) = 5.862; *p* < 0.05]. Bonferroni post-hoc analyses showed that KD-NO SA mice exhibited a significant decrease in CB1r gene expression in comparison with their corresponding SD group (*p* < 0.05) and the KD-SA group (*p* < 0.01). No significant differences were obtained in the gene expression of the opioid receptor mu (Figure 5f).

## 4. Discussion

The main aim of the present work was to evaluate whether a KD could modulate the rewarding and motivational effects of alcohol. In addition, the role in these diet-induced changes of receptors related to the reward process, such as adenosine, dopaminergic, cannabinoid, and opioid systems, were also considered through gene expression studies. The possible beneficial effects of nutrition as a complement to substance-use disorder treatments have been scarcely studied. Therefore, this work opens a new line of research in drug addiction, given that to date only unhealthy diets such as cafeteria, high-sugar, and high-fat diets have been investigated in this field (for review see [57,58]).

The results found in the present work revealed that the KD induces an overall decrease in alcohol SA but does not affect the motivation to get the drug. Furthermore, the KD by itself induces opposite changes in the gene expression of ADORA1 and CB1r, which disappeared after alcohol consumption. Moreover, KD after EtOH SA induced striatal increases in the gene expression of ADORA2, DrD1, and DrD2 genes. No changes in Oprm gene expression were observed. 

### 4.1. Ketogenic Diet Modulates Bodyweight and Increases β-Hydroxybutyrate Blood Levels

Animals in the KD group rapidly turned into a ketotic state and their βOHB levels stabilized after 7 days. The ketotic state was maintained throughout the experiment, even during EtOH SA, where a slight decrease in βOHB levels was observed. These could be due to a decrease of βOHB in the liver induced by alcohol, as it has been recently shown [59]. These authors showed that βOHB administration had an anti-inflammatory and hepatoprotective effect in mice with ethanol-induced liver disease, suggesting its possible therapeutic role for alcohol-use disorders. On the other hand, we have also observed a slight increase in βOHB levels in the control group as a result of food deprivation during EtOH SA, confirming that fasting also increases βOHB levels [60].

With respect to the bodyweight, our KD-exposed mice significantly increased their bodyweight in comparison to animals in the standard diet. Even though all groups experienced a decrease in bodyweight when SA food deprivation began (1 h/day access), the group exposed to KD maintained its weight above the SD group. To date, studies have shown conflicting results regarding the effects of KD on bodyweight. While some studies have found that KD for 2 weeks induces weight loss in rats [49], other studies show that bodyweight is increased by KD in rats when administered for 6 to 8 weeks, although the weight gain was slower than that observed in SD-fed animals [61,62]. Moreover, other studies reveal that male mice fed with a KD for a month showed an increase in brown adipose tissue (up to a 40% greater than controls) in agreement with our results [63].

### 4.2. Ketogenic Diet Diminishes Ethanol Self-Administration

Regarding EtOH SA, our results showed a general decrease in alcohol consumption in the KD group, both in FR1 and FR3 schedules. There were no differences in the number of effective responses between groups, meaning that animals on KD did not drink all the EtOH they had access to. This decrease in EtOH consumption was maintained across the ten days of self-administration, even during the FR3, where animals were demanded more work to obtain the drug.

Nevertheless, this decrease in alcohol intake did not modify the motivation to obtain the reward, as it was observed in the active responses and PR schedule, where no differences were observed between KD and SD. The PR indicates the breaking point or limit of active responses by the animals, that is, to what extent a mouse is willing to introduce its nose into the hole repeatedly to obtain one reinforcement (in this case, 38 µL 6% EtOH). Discarding an incorrect learning of the task (no differences in training or substitution), this result confirms that the mechanisms involved in FR1 and FR3 schedules (rates of operant responding) are not the same as those involved in PR (motivation) [64]. In fact, the PR schedule is complementary to the FR schedules and is indicative of the motivation for seeking the drug [65,66], while FR1 assesses the potential liability of a drug and the consumption based on its unconditioned psychopharmacological effects [67]. Moreover, these results support the fact that mice fed on KD do not show a motivational alteration towards reward.

To date, few studies have explored the relationship between alcohol consumption and KD, most of them focusing on ketoacidosis, a state in which the body’s insulin is so low that the liver produces excess levels of ketone bodies that would have neurotoxic properties [68]. Nevertheless, only a few studies have evaluated the interaction between KD and EtOH, one of them focused on the reduction of alcohol intake in rodents [51]. In this study, animals were first trained to lever press for alcohol in the vapor self-administration paradigm. Next, the rats were divided into two groups and exposed to a KD or SD for 8 weeks, after which both groups were returned to SD. Then, to induce alcohol dependence, half of the rats were exposed to chronic alcohol vapor for 7 weeks, and the other half, as a control group, were exposed to air. After this, animals were subjected again to a vapor self-administration paradigm. Their results showed that animals on KD-alcohol vapor did not exhibit an escalation in EtOH intake, as they self-administered EtOH to the same degree as non-dependent rats, suggesting that the KD exerted a protective effect. Regarding alcohol withdrawal symptoms, another study implemented a KD in rats before alcohol administration (intragastric administration) until the end of the procedure, although they did not verify whether the KD had increased ketone bodies [49]. Their results indicate that the KD was effective in reducing rigidity and irritability behaviors but the mechanism through which the KD had a positive effect on alcohol withdrawal symptoms remains unclear. Similar results have been obtained with administration of ketone monoester in mice [50], which reduces handling-induced convulsions and anxiety-like behaviors in early alcohol withdrawal, as with the KD.

Although the study focused on a psychostimulant drug, Martínez and co-workers [48] evaluated the potential of a KD as a therapy for cocaine addiction. The administration of a KD for three weeks to female and male rats was able to block cocaine sensitization, hyperlocomotion, and stereotypies, confirming a robust action of the KD on dopamine-mediated behaviors [48]. One of the explanations proposed by this study is that KD-mediated changes in adenosine may have therapeutic potential via actions at adenosine-dopamine receptor heterodimers.

Taking into account the different bodyweight of both groups, the results of EtOH consumption were calculated based on the individual weight of each subject (g/kg) in order for these differences to be avoided. A limitation of the present study could be the lack of control on the kcal intake between groups. Based on their bodyweight, we hypothesized that mice on KD ate a greater amount of kcal in an hour compared to the SD group during the SA procedure, indicating that the hypothesis of satiation as a contributor to the decrease in EtOH consumption should be taken into consideration. However, this possibility should be ruled out, as food intake occurred postprandially to SA, i.e., animals were introduced into the SA box 23 h after their last food intake. In addition, a recent study reported that food-deprived mice decreased their food intake after EtOH SA [69]. These results may explain why the SD group, which showed higher alcohol consumption, consumed fewer calories from food after consuming EtOH. In addition, food intake or satiation did not affect their EtOH motivation, as seen in the breaking point results. However, future studies should employ a eucaloric protocol to rule out this possible impact.

Nevertheless, even though studies report that a KD has beneficial effects, for example in weight loss in obese patients [70], the possible long-term effects of this diet remains unknown, probably due to the difficulty of adherence to a strict KD over time. Some studies have already reported that there can be adverse effects such as lipid abnormalities [71,72], hypoglycemia and dehydration [73,74], dysregulation of glucose levels [75,76] or nephrolithiasis [77]. Although some of these consequences have been seen in patients on the KD [77], the effects of a ketotic state in patients with an alcohol-use disorder may be pronounced, as they already present a poor nutritional status [78]. Thus, future studies should address this issue, considering the basic blood (cholesterol, low and high density lipoprotein, triglycerides, glucose) and liver parameters (transaminases) and investigate the possibility of using ketone esters as a supplement to a balanced diet, which also increase blood BHB [79,80].

### 4.3. Ketogenic Diet Diminishes Ethanol Intake through the Adenosine-Dopamine Binomial

Our initial hypothesis was that the administration of a KD would diminish the EtOH rewarding effects by acting through the adenosine-dopamine binomial. Our results showed that KD increased ADORA1 gene expression without affecting ADORA2 or dopaminergic genes. Therefore, KD induced an overexpression of the ADORA1 gene with regards to the DrD1 gene. When animals on KD were exposed to EtOH, the changes in gene expression were completely different. KD and EtOH exposure increased the expression of the DrD1, DrD2, and ADORA2 genes. Under these circumstances, there was an overexpression of the DrD1 gene with respect to the ADORA1 gene and no change of balance was observed with regard to ADORA2 and DrD2, as both genes increased their expressions.

The binomial refers to the mutual antagonistic interactions between the heterodimer’s A1-D1 and A2-D2 receptors [42]. Adenosine receptors are particularly expressed in GABAergic neurons in the striatum and are colocalized post-synaptically with dopamine receptors [41]. A1 agonists have been found to significantly decrease the binding affinity of D1, indicating that the function of the A1 receptor in the A1-D1 heterodimer is to inhibit dopamine signaling via the D1 receptor [81].

Although the activation of the A1 receptor has been demonstrated to be one of the main anti-seizure mechanisms of the KD [36], it seems that the A2 receptor collects more evidence in the addiction field, and this supports the results obtained in this study. For example, a mixed antagonism of both the A1 and A2 receptors, but with higher potency on the latter, produces similar effects for those generated by psychostimulants and enhances relapse into cocaine SA in baboons [82]. Likewise, A2 agonists decrease cocaine SA [83] and cocaine and morphine locomotor sensitization in rodents [46,84]. Finally, A2 knockout animals exhibit a significant decrease in cocaine SA, although conditioned place preference and sensitization were not affected [45,85]. 

There are also studies suggesting that adenosine mediates EtOH intake as well. Confirming our results, Feltmann and co-workers [86] reported an increase in the density of the A2-D2 heteroreceptor in the striatum of rats that voluntarily drank alcohol for 12 weeks, although a reduction of the striatal density of D2-D2 homoreceptor complexes was also observed. In addition, A2 receptor stimulation with A2 agonists attenuated alcohol intake, while the A2 antagonist increased EtOH intake in alcohol-preferring rats [87] and mice [88].

Moreover, studies about KDs and dopaminergic activity are scarce. However, it has been reported that 3 weeks of KD does not change DA in the Nucleus Accumbens [89]. A possible explanation for the decreased EtOH SA observed in mice on KD could be the relative increase of the ADORA1 gene, which in turn inhibits DrD1. With respect to the heterodimers A2-D2, KD did not induce any changes but both genes were overexpressed after oral EtOH SA, which indicates that there is no change in the balance of these genes.

As we mentioned, the GABAergic system is one of the most likely targets of EtOH [90] in addition to ketone bodies [91]. KD induces an increase in GABA-mediated inhibition and activates the GABA-B receptors [11,92]. Results showing the efficacy of GABA-B agonists decreasing EtOH SA [93,94,95] sustain the hypothesis that a KD modulates the GABAergic system through a change in the adenosine-dopamine heterodimer. Taking into account that both EtOH and KD interact with the GABAergic system and produce alterations in the adenosine-dopamine heterodimer, we can hypothesize that ketone bodies could induce a decrease in EtOH consumption through this mechanism.

The cannabinoid and opioid systems are both involved in fatty food intake and reward [4,5]. In this study we observed that the KD induced a significant underexpression of the CB1 gene, but after EtOH consumption, this underexpression was normalized. Calorically dense foods increase CB1r density in the Nucleus Accumbens, leading to their downregulation [96], which could be the case for KD. Moreover, it is important to note that most GABAergic inhibitory interneurons express presynaptic CB1r in abundance, which modulate the release of GABA at the synapses [97,98]. Regarding Oprm, this study reported no significant changes except for a trend to be overexpressed in the KD groups. Some studies suggest that Oprm presents an increase of expression in the brain areas processing reward associated with palatable foods [99,100], but there are no studies about the effects of KD on the opioid system.

## 5. Conclusions

KD may be a useful nutritional complement to the existing pharmacological therapies in alcohol addiction, especially considering the undernourishment that alcohol produces. In addition, we propose the adenosine-dopamine binomial as an interesting target that deserves to be explored in alcohol addiction. One important limitation of the KD is the difficulty to maintain adherence over time, and a permanent ketotic state is not considered realistic as a way of life. Stemming from this, there is a wide range of research that can be done, including testing other ketogenic-related therapies such as ketone esters, which promote an increase in blood βOHB levels. Furthermore, even when our results confirm that a KD could have a positive effect on reducing alcohol intake in mice, further research is needed to know the long-term effects of KD on metabolic health, such as basic blood and liver parameters. To date, although there is a scarce number of published studies highlighting the protective effects of KD on drug effects, the results obtained have been reported in preclinical studies, therefore more studies assessing the effect of different ketogenic diets on addiction in humans are needed. Many studies remain to be carried out with KD, and it is necessary to elucidate the exact neurobiological mechanisms through which KD modulates addiction. Even so, these results highlight once again the great relevance of nutritional interventions in mental and substance-use disorders.

## Data Availability

Data is contained within the article.
